# Persistence of Positive Carryover Effects in the Oyster, *Saccostrea glomerata*, following Transgenerational Exposure to Ocean Acidification

**DOI:** 10.1371/journal.pone.0132276

**Published:** 2015-07-06

**Authors:** Laura M. Parker, Wayne A. O’Connor, David A. Raftos, Hans-Otto Pörtner, Pauline M. Ross

**Affiliations:** 1 School of Science and Health, University of Western Sydney, Hawkesbury K12, Locked Bag 1797, Penrith South DC 2751, Sydney, New South Wales, Australia; 2 NSW Department of Primary Industries, Port Stephens Fisheries Centre, Taylors Beach, New South Wales, Australia; 3 Department of Biological Sciences, Macquarie University, North Ryde, New South Wales, Australia; 4 Alfred Wegener Institute for Polar and Marine Research in the Hermann von Helmholtz Association of National Research Centres e. V. (HGF), Am Handelshafen 12, Bremerhaven, Germany; University of Hong Kong, HONG KONG

## Abstract

Ocean acidification (OA) is predicted to have widespread implications for marine organisms, yet the capacity for species to acclimate or adapt over this century remains unknown. Recent transgenerational studies have shown that for some marine species, exposure of adults to OA can facilitate positive carryover effects to their larval and juvenile offspring that help them to survive in acidifying oceanic conditions. But whether these positive carryover effects can persist into adulthood or the next generation is unknown. Here we tested whether positive carryover effects found in larvae of the oyster, *Saccostrea glomerata* following transgenerational exposure to elevated CO_2_, could persist into adulthood and whether subsequent transgenerational exposure of adults to elevated CO_2_ would facilitate similar adaptive responses in the next generation of larvae and juveniles. Following our previous transgenerational exposure of parental adults and first generation (F1) larvae to ambient (385 μatm) and elevated (856 μatm) CO_2_, newly settled F1 juveniles were transferred to the field at ambient CO_2_ for 14 months, until they reached reproductive maturity. At this time, the F1 adults were returned to the laboratory and the previous transgenerational CO_2_ exposure was repeated to produce F2 offspring. We found that the capacity of adults to regulate extracellular pH at elevated CO_2_ was improved if they had a prior history of transgenerational exposure to elevated CO_2_. In addition, subsequent transgenerational exposure of these adults led to an increase in the resilience of their larval and juvenile offspring. Offspring with a history of transgenerational exposure to elevated CO_2_ had a lower percentage abnormality, faster development rate, faster shell growth and increased heart rate at elevated CO_2_ compared with F2 offspring with no prior history of exposure to elevated CO_2_. Our results suggest that positive carryover effects originating during parental and larval exposure will be important in mediating some of the impacts of OA for later life-history stages and generations.

## Introduction

Near-future ocean acidification threatens the survival of marine organisms and the ecosystems they support [1]. With atmospheric carbon dioxide (CO_2_) emissions continuing to increase at an unprecedented rate, the likelihood of alleviation through mitigation efforts is low [2–3]. As a consequence, many marine species will need to undergo rapid acclimation or adaptation to avoid extinction [2–6]. Accordingly, we are currently seeing a shift in the focus of ocean acidification research internationally, from studies which simply measure the acute response of marine organisms to ocean acidification, most typically within a single life-history stage or single generation, to those that test for the capacity of species to acclimate or adapt to the acidifying conditions [5,7–18].

Transgenerational experiments are one area of investigation which are currently gaining considerable ground in the literature [5,10,13,16,17–18]. These experiments expose parents to elevated CO_2_ during reproductive conditioning and measure how this parental exposure influences the performance of their offspring. For most species studied to date, exposure of parents to elevated CO_2_ has facilitated the transfer of positive carryover effects (*transgenerational effects*) to larval and juvenile offspring that have helped the offspring survive in the acidified conditions [5,10,13] (but not Welch et al. [17]). For example, Miller et al. [10] found that juvenile anemone fish, *Amphiprion melanopus*, from parents with no previous exposure to elevated CO_2_ had increased metabolic rate and decreased weight and length when exposed to elevated CO_2_ of 1000 μatm. These negative effects were no longer present and in some cases were reversed, however, when their parents were also exposed to elevated CO_2_.

Yet, a limitation of nearly all transgenerational experiments to date is that researchers only consider the response of an organisms’ early life-history stages (larvae and juveniles), with no consideration of how the future adult will respond. Ontogenetic stages of marine organisms differ considerably in their forms and functions and have various degrees of sensitivity to environmental stressors [19]. As such, the phenotypic traits which benefit early life-history stages during exposure to elevated CO_2_ may not necessarily benefit other life-history stages (i.e. adults) [20]. In addition, we have little understanding of the longevity of positive carryover effects in marine organisms. Are these effects short-lived and present only in the early life-history stages? Do they persist into adulthood? If so, what are the implications of this for the next generation? Studies on non-marine vertebrates showed that the conditions experienced by an organism in early life can have direct effects on their reproductive success later as well as the success of future generations (Burton and Metcalfe [21] and references therein). Understanding whether adaptive responses obtained in the early life-history stages can carryover to influence the fitness and survival of adults and the next generation during exposure to elevated CO_2_ remains a significant challenge and knowledge gap, particularly in organisms with long generation times.

A recent transgenerational study on the three-spined stickleback *Gasterostreus aculeatus* showed that the persistence level of transgenerational carryover effects during the juvenile stage was trait specific [16]. For example, parental exposure to elevated CO_2_ led to an increase in the size (influenced by paternal exposure but not maternal) and area of otoliths of *G*. *aculeatus* juveniles that were reared at both ambient and elevated CO_2_ but caused a reduction in the survival and growth of juveniles that were reared at ambient CO_2_. The transgenerational effects on otolith characteristics were still present 100 days post-hatch whereas the effects on survival and growth persisted for only 40 days post-hatch. Following this time, strong within-generational impacts were found on survival and growth, suggesting that both trans- and within-generational impacts have the capacity to shape the response of later life-history stages and potentially future generations to ocean acidification. In the only study to date to measure the transgenerational response of elevated CO_2_ on adults, Thor and Dupont [17] found that elevated CO_2_ caused a significant reduction in the fecundity of adults of the calanoid copepod, *Pseudocalanus acuspes*, with no previous history of exposure to elevated CO_2_. But this effect was partially alleviated in adults that experienced transgenerational exposure to elevated CO_2_ over multiple generations, suggesting that positive carryover effects can indeed persist in adults following transgenerational exposure. However, this species has a relatively short generation time (approx. 10 weeks to reach reproductive maturity). Whether similar positive carryover effects can persist in species with longer generation times remains to be determined.

The Sydney rock oyster, *Saccostrea glomerata* is an iconic mollusc species found inhabiting hard substratum in estuarine locations on the southeast coast of Australia where it forms the basis of the edible oyster industry [22]. Moreover, it has a relatively long generation time, taking approximately 9–12 months to reach reproductive maturity. Acute studies show that early life-history stages of *S*. *glomerata* are extremely vulnerable to elevated CO_2_ [23–26], however, these effects are partially alleviated when their parents are reared at the same level of CO_2_ during reproductive conditioning [5]. Larvae from parents conditioned at elevated CO_2_ were larger in size and developed faster at both ambient and elevated CO_2_ when compared with larvae from parents conditioned at ambient CO_2_. The question remaining for *S*. *glomerata* is whether these positive carryover effects are limited to larval development or whether they can persist into adulthood and the next generation.

Herein, we extend our investigation to the first generation (F1) adults and second generation (F2) larvae and juveniles to test whether the positive carryover effects found in larvae following the previous parental transgenerational exposure can persist and be beneficial in adulthood. Moreover, we investigate whether similar positive carryover effects can be passed from F1 adults to F2 larvae and juveniles following subsequent transgenerational exposure. While this experiment tests whether positive carryover effects are passed to F1 adult *S*. *glomerata* following transgenerational exposure to ocean acidification, these effects may have arisen during the parental exposure, F1 larval development or a combination of the two. For this reason, throughout this study when referring to the F1 adults, we use the term ‘carryover effects’ to describe changes in physiological traits of the F1 adults that have occurred due to transgenerational effects (carryover effects passed from parents to offspring i.e. nutritional, somatic, cytoplasmic, epigenetic modifications or genetic selection) and/or within-generational effects (carryover effects which originated during larval development i.e. phenotypic plasticity or genetic selection)[2–3,21].

## Materials and Methods

In 2010, the experiment began by purchasing 300 wild caught adult Sydney rock oysters from an oyster farm in Port Stephens, New South Wales (NSW) (32°45’S, 152°10’E) Australia (Holbert’s Oyster Supplies). These adults made up the ‘parental generation’ and were approximately 1.5–2 y of age at the time of collection and were at the early stages of gametogenesis; mean whole weight = 52.35 ± SE 1.51 g). The parental generation oysters were transferred in hessian bags to the Department of Primary Industries (DPI) Port Stephens Fisheries Institute (PSFI), Taylors Beach, NSW, Australia. Once in the laboratory the adults were scrubbed to remove mud and fouling organisms and were transferred into 40 L tubs which were supplied with 1 μm nominal filtered recirculating seawater from 750 L header tanks (2 tubs per header tank; 24°C; salinity 34.6 ppt), fed an algal diet of 50% *Chaetoceros muelleri*, 25% *Pavlova lutheri* and 25% Tahitian *Isochrysis aff*. *galbana* at a concentration of 2 x 10^9^ cells oyster^-1^ day^-1^ and allowed to acclimate to laboratory conditions for 2 w.

The experiment was divided into 2 transgenerational exposure periods: *1*. *Parental generation adults to First generation (F1) larvae; 2*. *F1 adults to Second generation (F2) larvae and juveniles*.

### Parental generation adults to F1 larvae

**Fig 1:**
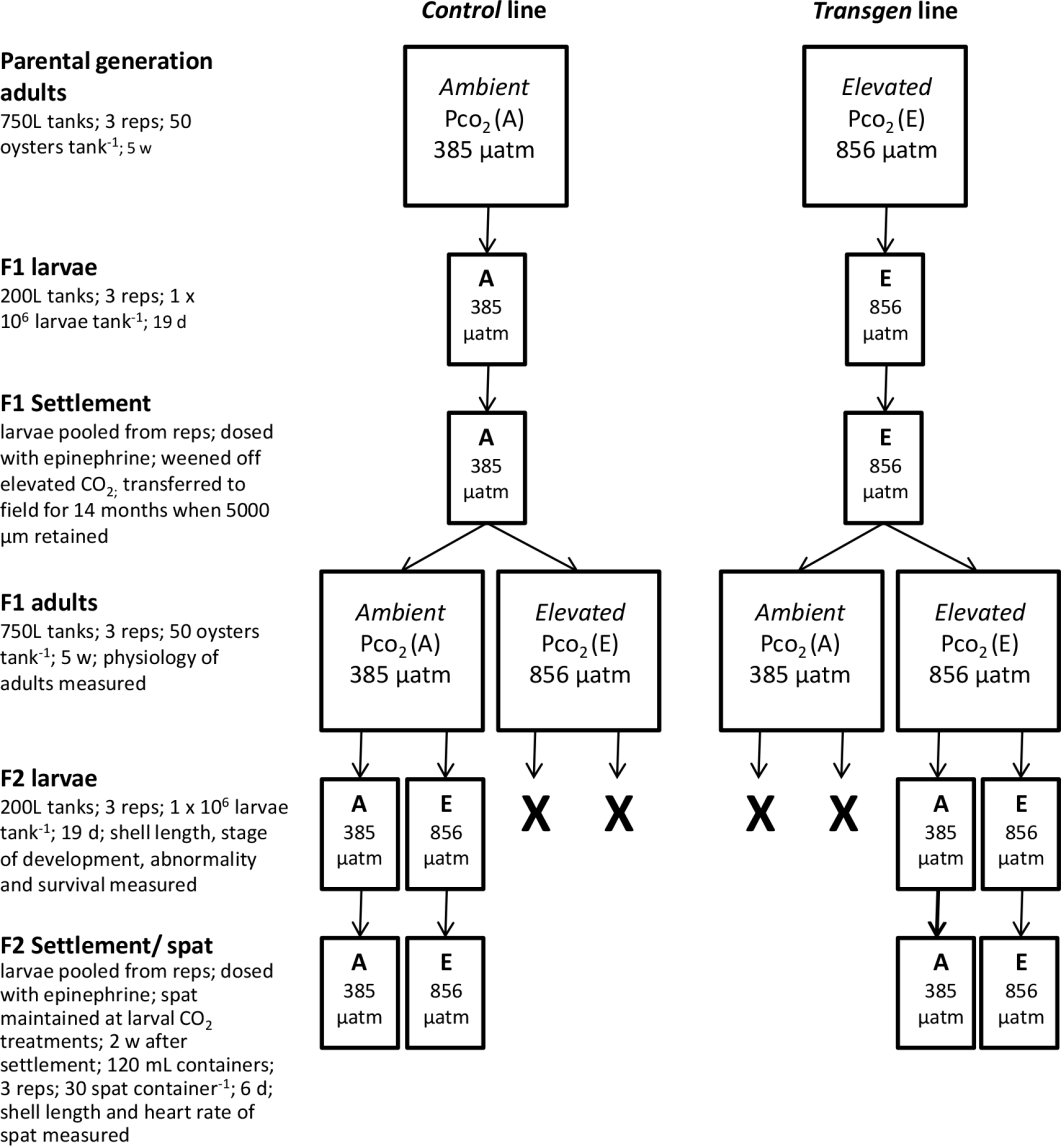
Flow chart of the experimental design. X indicates that these lines were not followed through to the F2 generation.

For detailed information on the first transgenerational exposure (Parental generation adults to F1 larvae) see Parker et al. [5]. Briefly, following the 2 w acclimation period, parental generation adults were divided at random into 6 groups of 50 oysters. Each group was transferred into a separate 40 L tub supplied by its own 750 L header tank. Three of the header tanks were set at ambient CO_2_ of 385 μatm while the other three were set at elevated CO_2_ of 856 μatm. Adults were fed the same combined algal diet provided during their acclimation period. Complete water changes were made every 2 d using preequilibrated filtered seawater (FSW) and oysters were briefly removed out of water each day (10 seconds) and rinsed with freshwater to remove faecal matter. Following 5 w of conditioning in the treatments the adults reached gravid stage and were strip spawned to allow the collection of eggs and sperm. Eggs and sperm from a minimum of 10 females and 10 males from each ‘parental adult CO_2_ treatment’ (ambient or elevated CO_2_) were filtered to remove debris and were fertilised in 20 L buckets at the same CO_2_ level that the adults were held at (FSW, 24°C, salinity 34.6 ppt). The gametes were allowed to fertilise for 30 min to create two F1 offspring lines: ambient parental adult exposure, ambient F1 larval exposure (referred to here after as the *‘*F1*-control* line’); and elevated parental adult exposure, elevated F1 larval exposure (referred to here after as the *‘*F1*-transgen* line’). Following fertilisation the embryos from each line were transferred into 200 L polyethylene larval rearing tanks held at the same CO_2_ concentration that they were fertilised in. There were three replicates for each of the two offspring lines (Fig 1). Larval feeding began after 16 h with the appearance of the first D-veliger larval stage. Larvae were fed an algal diet twice daily consisting of 50% *Chaetoceros calcitrans*, 25% *P*. *lutheri* and 25% T. *Isochrysis aff*. *galbana* for the first week of development [27]. After this time, *C*. *calcitrans* was gradually replaced with *C*. *muelleri* as the larvae increased in size. Algal concentrations ranged from 1 x 10^4^ cells mL^-1^ at the beginning of the larval experiment up to 1.16 x 10^5^ cells mL^-1^ at the completion of the larval experiment. Once the larvae reached eyed stage and showed signs of settlement (eye spot, 300 μm shell length, protruding foot, crawling, approximately 19 d– 21 d depending on the larval and adult CO_2_ exposure treatment; see O’Connor et al. [27]) each replicate line was dosed with epinephrine (60 mg epinephrine per 2.5 g of larvae in 1 L FSW) to help induce settlement and were suspended on a 180 μm mesh settlement screen [27]. This is a common practice used in the hatchery production of this species [27]. To ensure maximum settlement and representation of each line, this process was repeated every 2 d for 6 d. Newly settled juvenile spat were rinsed with freshwater and brushed gently daily to prevent them from settling on the screens.

Following settlement, the newly metamorphosed spat were slowly weaned off the elevated CO_2_ treatment over a 2 w period (0.05 pH unit increase every 2 d) and replicates of each treatment were pooled. Spat were fed an algal diet of 50% *C*. *muelleri*, 25% *P*. *lutheri* and 25% T. *Isochrysis aff*. *galbana* at a concentration of 50,000 cells spat^-1^ d^-1^ and remained in the laboratory under ambient conditions until they were large enough to be transferred into the field (approx. 5000 μm retained). Once the spat were large enough they were transferred in oyster bags back to the estuary at Port Stephens, NSW, Australia where they remained for a period of 14 mo until they reached reproductive maturity. By growing the F1 oysters from juveniles to adults in a common-garden of ambient CO_2_ conditions for 14 months it allowed us to reset the environmental history of the oysters and determine whether any physiological improvements seen in the F1-*transgen* adults during subsequent exposure to elevated CO_2_ occurred due to the transgenerational adult and larval exposure and not due to phenotypic plasticity of later life-history stages.

### F1 adults to F2 larvae and juveniles

Upon reaching reproductive maturity 300 adult oysters from each of the two F1 offspring lines (F1-*control* and F1-*transgen* line) were returned from the common-garden of ambient CO_2_ in the field, to the laboratory. Once in the laboratory they were cleaned to remove mud and fouling organisms and acclimated identically to the parental generation for 2 w (mean whole weight = F1-*control*: 25.89 ± SE 1.25g; F1-*transgen*: 26.00 ± SE 1.53 g). Following the acclimation period each line was divided at random into 6 groups of 50 oysters and were transferred into separate 40 L tubs. There were 12 40L tubs altogether which were supplied with recirculating FSW from 6 750 L header tanks. Each header tank supplied one 40 L tub of F1*-control* and one 40 L tub of F1-*transgen* oysters. Using a fully orthogonal design, three of the header tanks were set at ambient CO_2_ (385 μatm) and three were set at elevated CO_2_ (856 μatm). The adults were fed and maintained identically to the previous parental generation experiment and remained in the treatments for 5 w until they reached gravid stage. At this time (5 w), 2 oysters were removed from each replicate tub and the standard metabolic rate (SMR) and extracellular pH (pH_e_) of the F1 adults was measured. The remaining adults were removed from the 40L tubs in preparation for spawning to create the F2 offspring. For more details on the experimental design see Fig 1.

#### Determination of extracellular pH (pH_e_) and SMR in F1 Adults

To determine the pH_e_ of the F1 adult oysters, hemolymph was removed by pericardial puncture using a Hamilton syringe and transferred into a microcentrifuge tube (0.5 mL) for immediate analysis. pH_e_ was determined using a Metrohm micro pH meter calibrated using NIST buffers. SMR in the F1 adults was determined using a closed respirometry system according to Parker et al. [5]. Feeding was stopped 24 h prior to the measurements. Oysters were then placed in 500 mL airtight chambers which were fitted with a fibre-optic probe (PreSens dipping probe DP-PSt3, AS1 Ltd, Palmerston North, New Zealand) calibrated using a two-point calibration (0% and100%) at 24°C. The oysters remained in the chambers to record the time taken for them to reduce the oxygen concentration in the chamber from 100% to 80%. At this time, the oysters were removed and shucked to determine their dry tissue mass. SMR was calculated as follows:

$$SMR=\frac{V_{r}(L)\times \Delta C_{w}O_{2}(mgO_{2}L^{-1})}{\Delta t(h)\times bw(g)}$$

where SMR is the oxygen consumption normalized to 1 g of dry tissue mass (mg O_2_ g^-1^ dry tissue mass h^-1^, V_r_ is the volume of the respirometry chamber minus the volume of the oyster (L), ΔC_w_O_2_ is the change in water oxygen concentration measured (mg O_2_L^-1^), Δ*t* is measuring time (h) and bw is the dry tissue mass (g) of the oyster. Six oysters were measured from each F1 line (*Control* or *Transgen*) and F1 CO_2_ treatment (Ambient or Elevated CO_2_) combination (2 oysters from each replicate tank_;_ n = 3).

#### F2 Larval development

Two F1 lines were used to create the F2 generation: *Control line*–oysters that were held at ambient CO_2_ for parental and F1 larval and adult exposure; *Transgen line*—oysters that were held at elevated CO_2_ for parental and F1 larval and adult exposure. Eggs and sperm from a minimum of 10 females and 10 males from each F1 line (F1-*control* or F1-*transgen*) were filtered to remove debris and were fertilised in 20 L buckets (FSW, 24°C, salinity 34.6). There were two buckets for each line, with one bucket filled with ambient CO_2_ FSW (385 μatm) and the other filled with elevated CO_2_ FSW (856 μatm). The gametes were divided equally across the buckets and allowed to fertilise for 30 min to create four F2 offspring lines: 1) ambient parental exposure, ambient F1 exposure, ambient F2 exposure (referred to hereafter as the F2 *control* at ambient CO_2_); 2) ambient parental exposure, ambient F1 exposure, elevated F2 exposure (referred to hereafter as the F2-c*ontrol* at elevated CO_2_); 3) elevated parental exposure, elevated F1 exposure, ambient F2 exposure (referred to hereafter as the F2-*transgen* at ambient CO_2_); 4) elevated parental exposure, elevated F1 exposure, elevated F2 exposure (referred to hereafter as the F2-*transgen* at elevated CO_2_). The resulting gametes were transferred into 3 replicate 200 L tanks held at the same seawater conditions that they were fertilized in. They remained in these treatments for the duration of larval development (19 d until they reached the eyed stage). At 2 d intervals, starting at day 1, a subsample was removed from each replicate larval tank and 10 larvae tank^-1^ were observed under the microscope (Leica 100X, Wetzlar, Germany). The shell length (antero-posterior measurement) of larvae was measured at each 2 d interval, percentage of abnormal larvae (D-veligers only) was measured after 1 d and the percentage of eyed larvae and percentage survival of larvae was determined after 19 d.

#### F2 Juvenile Spat

In order to test the impacts of elevated CO_2_ on F2 juveniles, larvae that showed signs of being ready to settle and metamorphose, were induced to set by dosing with epinephrine using the same procedure used in the previous F1 larval exposure experiment. Newly settled spat were suspended on 180 μm settlement screens and were rinsed with freshwater and brushed gently daily to prevent them from settling on the screens. The spat from each line remained in the same CO_2_ exposure treatment that they were reared in for larval development and were fed the same combined algal diet as the larvae at a concentration of 50,000 cells spat^-1^ d^-1^. Water changes were made every 2 d. Two weeks after settlement, 90 spat were removed from each line, and divided into 3 groups of 30 spat. The shell length of each spat was measured under the light microscope (Leica 100, Wetzlar, Germany) before being transferred into three 120 mL containers with screw capped lids (30 spat container^-1^). The CO_2_ level in the 120 mL containers was identical to the level that the spat were already being held in and was obtained by direct bubbling of CO_2_ to the desired pH. Spat were fed the same mixed algal diet at a concentration of 50,000 cells spat^-1^ d^-1^ and complete water changes were made daily. After 6 d in the treatments the shell length of each spat was once again measured. The impact of CO_2_ and transgenerational exposure on the growth of spat was determined by subtracting the mean shell length of spat at the beginning of the experiment with the mean shell length of spat at the end of the experiment (30 spat measured from each replicate line under the light microscope; Leica 100X, Wetzlar, Germany). We also measured the impact of elevated CO_2_ and transgenerational exposure on the heart rate of spat. Ten spat from each replicate line were placed on a petri dish in FSW at the same CO_2_ treatment that they were being held and were viewed under a dissecting microscope ensuring that the heart was visible. For each spat, the time taken for the heart to beat 20 times was recorded. This was then converted to the number of beats per minute (BMP) using the formula: (20*BPM / Time*[sec])×60sec.

### Manipulation of carbonate chemistry

The elevated CO_2_ concentration was obtained in each experiment by manipulation of pH by direct bubbling of CO_2_ in seawater controlled by independent pH negative feedback systems (Aqua Medic; accuracy ±0.01). Each negative feedback system consisted of a computer connected to a pH probe (calibrated daily using NIST buffers) and solenoid valve. When pH rose above the desired level, the solenoid valve would open and dose the tank with CO_2_. For the adult exposure experiments, the pH was constantly controlled in each 750 L header tank. For the larval and spat exposure, pH was adjusted in the 200 L and 120 mL tanks, respectively, at each water change and the tanks were sealed to minimize gas exchange (see Parker et al. [5,25]). The pH_NIST_ level was measured twice daily using a combined pH electrode. To determine the pH corresponding to the desired CO_2_ level the pH, total alkalinity (TA), temperature and salinity of the seawater was entered into the CO_2_sys program developed by Lewis and Wallace [28] using the dissociation constants of Mehrbach et al. [29]. TA was measured in triplicate before and after each water change by Gran titration (mean TA_F1 Adults_ = 2334 ± SE 48 μmol kg^-1^; mean TA_F2 Larvae_ = 2329 ± SE 41 μmol kg^-1^; mean TA_F2 Spat_ = 2333 ± SE 42 μmol kg^-1^; see Table 1 for seawater physiochemical conditions).

**Table 1:** Mean seawater physiochemical conditions during the F1 adult and F2 larval and spat CO_2_ exposure experiments.

	Condition	Salinity	Temperature (°C)	pH_NBS_	TA (μmol kg^-1^)	Pco_2_ (μatm)	DIC (μmol kg^-1^)	Ω_aragonite_	Ω_calcite_
*F1 Adults*	Ambient	34.5 ± 0.3	24.0 ± 0.5	8.20 ± 0.01	2334 ± 48	385	2032.2	5.22	3.43
	Elevated	34.5 ± 0.3	24.0 ± 0.5	7.90 ± 0.01	2334 ± 48	856	2181.0	2.98	1.95
*F2 Larvae*	Ambient	34.5 ± 0.2	24.0 ± 0.5	8.20 ± 0.01	2329 ± 41	385	2028.2	5.20	3.41
	Elevated	34.5 ± 0.2	24.0 ± 0.5	7.90 ± 0.02	2329 ± 41	856	2176.5	2.96	1.95
*F2 Spat*	Ambient	34.5 ± 0.2	24.0 ± 0.5	8.20 ± 0.03	2333 ± 42	385	2031.4	5.21	3.42
	Elevated	34.5 ± 0.2	24.0 ± 0.5	7.90 ± 0.05	2333 ± 42	856	2180.1	2.97	1.95

Values for Pco_2_, DIC, Ω_aragonite_ and Ω_calcite_ calculated from salinity, temperature, pH_NIST_ and TA. TA, total alkalinity; DIC, dissolved inorganic carbon; TA ± SE

### Data analysis

To determine any significant differences between ‘CO_2_ treatment’, and ‘transgenerational exposure’ on F1 adults of *S*. *glomerata*, pH_e_ and SMR of adults were analysed using a three-way Analysis of Variance (ANOVA) using GMAV5 [30], where ‘CO_2_ treatment’ and ‘transgenerational exposure’ were fixed and orthogonal factors and ‘tank’ was random and nested in CO_2_ treatment.

To determine any significant differences between ‘CO_2_ treatment’ and ‘transgenerational exposure’ on the F2 larvae and spat of *S*. *glomerata*, the percentage survival, percentage development and shell length (anteroposterior measurement) of eyed larvae after 19 d, percentage of abnormal larvae after 1 d and the shell growth and heart rate (BPM) of spat were analysed using a two-way ANOVA, where ‘CO_2_ treatment’ and ‘transgenerational exposure’ were fixed and orthogonal factors. Cochran's test was used to determine any heterogeneity of variances and data were transformed if significant. A Student Newman Keuls (SNK) test was used to detect differences amongst means [31].

## Results

### F1 Adults

**Fig 2:**
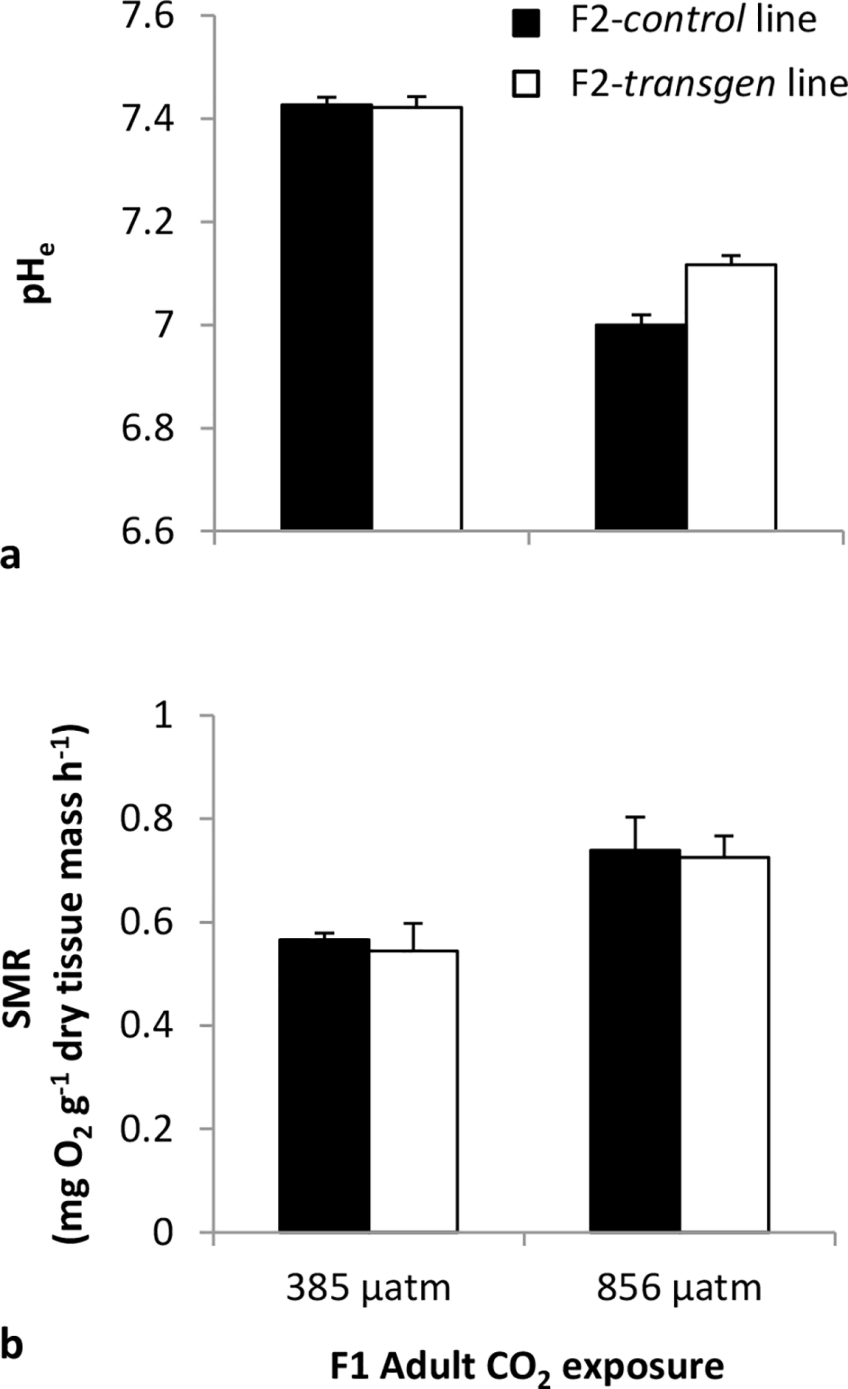
The response of F1-*control* and F1-*transgen Saccostrea glomerata* adults to ambient and elevated CO_2_. Extracellular pH (pH_e_) **(a)**, standard metabolic rate (SMR) **(b)** after 5 w of exposure to the CO_2_ treatments (mean ± SE) (24°C, salinity 34.5 ppt).

There was a significant interaction effect between CO_2_ and transgenerational exposure on the pH_e_ of F1 adults (Fig 2a; MS = 0.02, F = 25.26, df = 1; *P*< 0.01). After 5 w at elevated CO_2_, the F1-*control* adults experienced a reduction in pH_e_ (-0.43 pH unit reduction compared with the F1-*control* adults reared at ambient CO_2_). In the F1-*transgen* adults, there was also a reduction in pH_e_ after 5 w at elevated CO_2_, however, the reduction was to a significantly lesser degree (-0.3 pH unit reduction compared with the F1-*transgen* adults reared at ambient CO_2_) (Fig 2a; MS = 0.02, F = 25.26, df = 1; *P*< 0.01; SNK 385 μatm: F1-*transgen* = F1-*control*, 856 μatm: F1 CO_2_-exposed > F1 control).

There was a significant effect of elevated CO_2_ but not transgenerational exposure on the whole organism SMR of the F1 adults (Fig 2b; MS = 0.19; F = 132.96; df = 1, *P*< 0.001). Exposure to elevated CO_2_ for 35 d led to an increase in SMR in both F1 adult lines (Fig 2b). But the extent of this increase was similar in both the F1-*control* and F1-*transgen* adults.

### F2 larvae and spat

#### Survival of larvae

**Fig 3:**
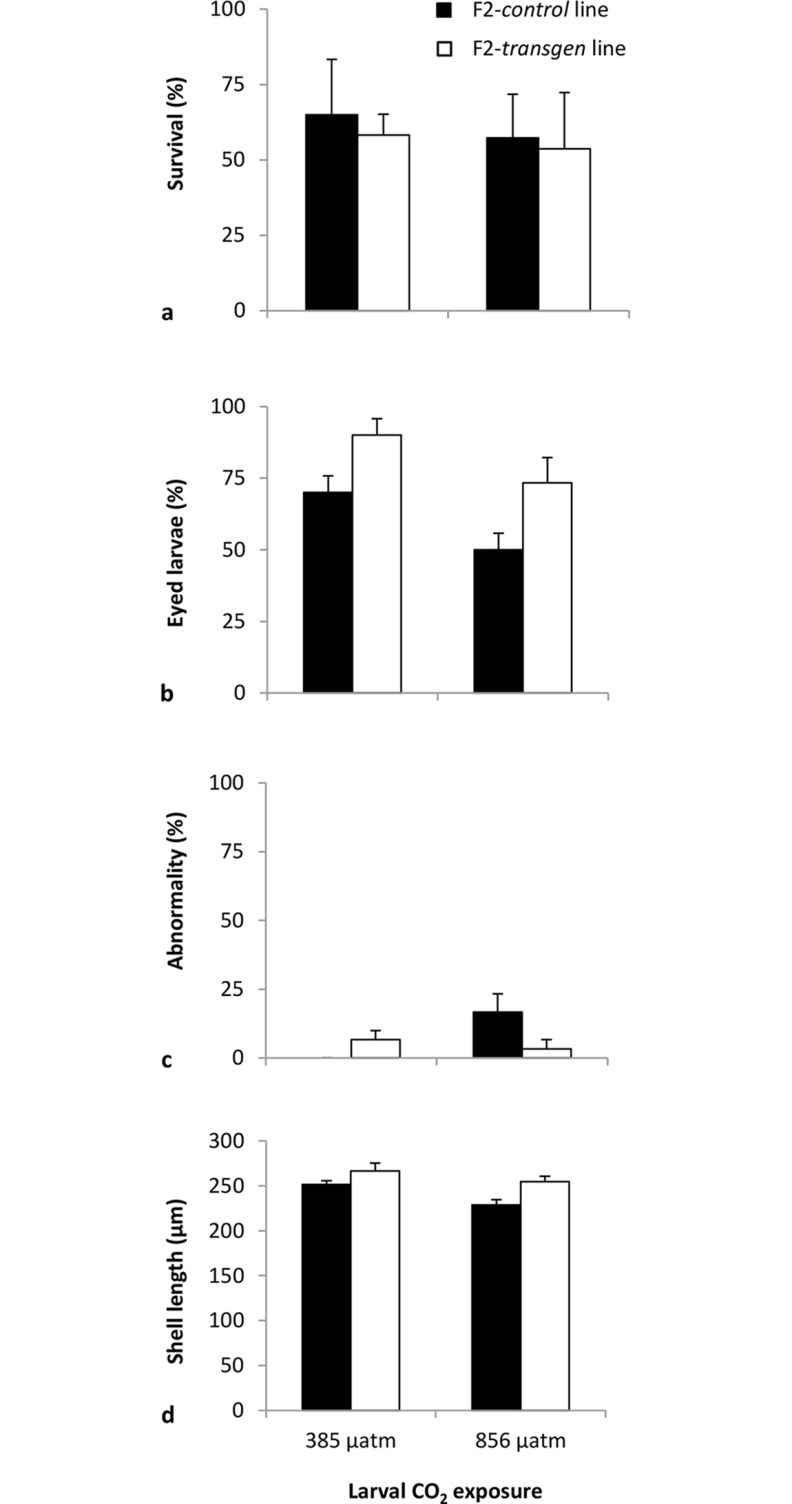
The response of F2-*control* and F2-*transgen Saccostrea glomerata* larvae to ambient and elevated CO_2_. Percentage survival of larvae after 19 d **(a)**, percentage eyed larvae after 19 d **(b)**, percentage abnormality after 1 d **(c)**, shell length after 19 d **(d)** of exposure to the CO_2_ treatments (mean ± SE) (24°C, salinity 34.5 ppt).

There was no effect of elevated CO_2_ or transgenerational exposure on the survival of F2 larvae (Fig 3a). After 19 d, the percentage survival of larvae was similar at ambient (385 μatm) and elevated (856 μatm) CO_2_ in both F2 lines (F2-*control* larvae and F2-*transgen* larvae; Fig 3a; MS = 7.13, F = 0.01, df = 1 x 8).

#### Development of larvae

There was a significant effect of elevated CO_2_ and transgenerational exposure but no interaction, on the number of larvae which developed to the eyed larval stage after 19 d in the treatments (Fig 3b). Both F2 larval lines experienced an increase in development time following exposure to elevated CO_2_ as depicted by a decrease in the percentage eyed larvae compared with those reared at ambient CO_2_ (Fig 3b; MS = 1008.33, F = 7.56, df = 1 x 8, P< 0.05). The development rate of the F2-*transgen* larvae, however, was significantly greater than the F2-*control* larvae (Fig 3b; MS = 1408.33, F = 10.56, df = 1 x 8, P< 0.05). Overall, after 19 d at elevated CO_2_, 73% of the F2-*transgen* larvae had reached the eyed larval stage (final larval stage before settlement), compared to only 50% in the F2-*control* larvae (Fig 3b).

There was a significant interaction effect between elevated CO_2_ and transgenerational exposure on the number of abnormal larvae which developed after 1 d (Fig 3c). At ambient CO_2_, the number of abnormal larvae was similar between the two F2 lines. At elevated CO_2_, however, there was an increase in abnormal development in the F2-*control* larvae but not in the F2 *CO*_*2*_ *-transgen* larvae (Fig 3c; MS = 300.00, F = 6.00, df = 1 x 8, *P*< 0.05).

#### Shell length of larvae

**Fig 4:**
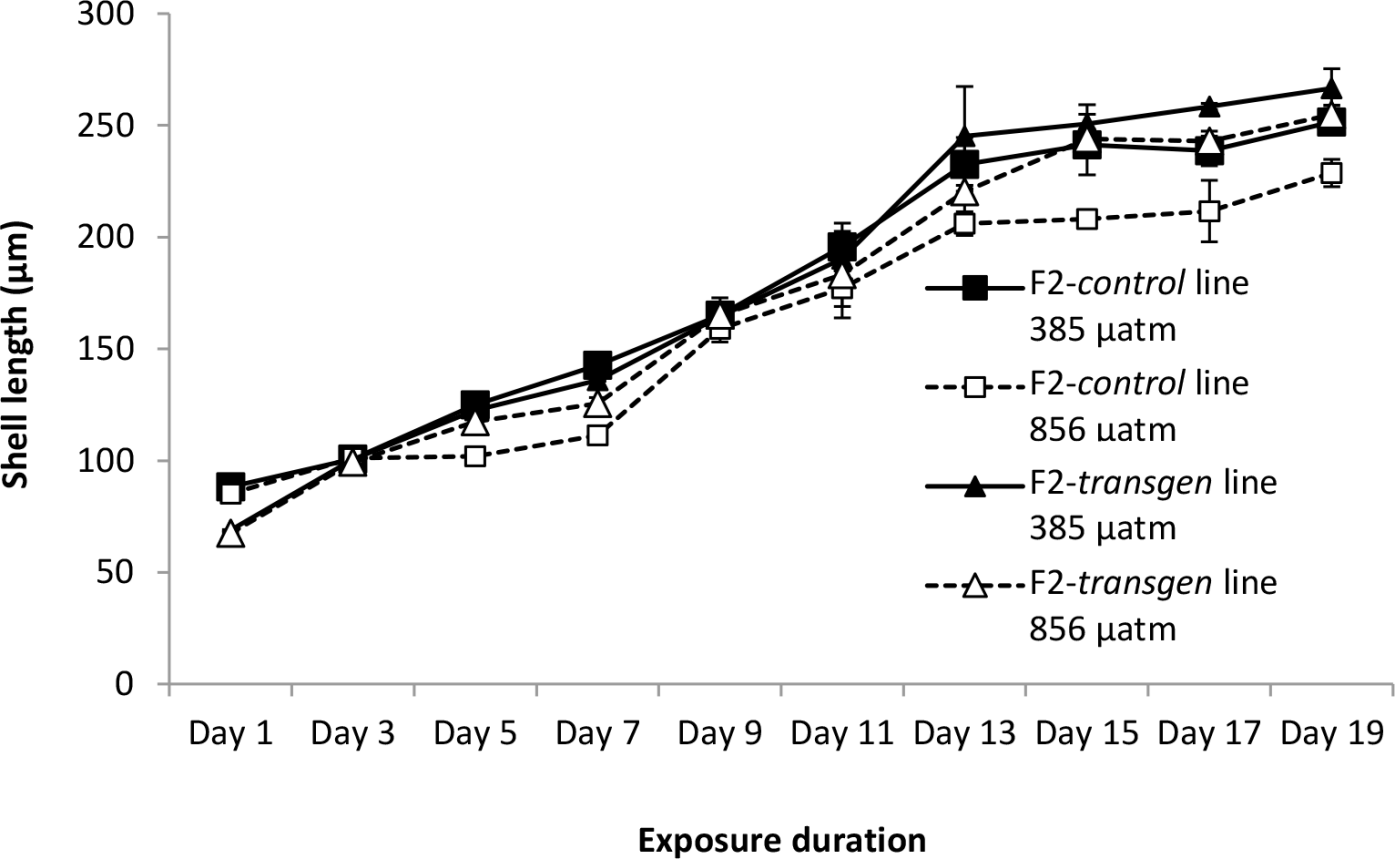
Shell length of F2-*control* and F2-*transgen Saccostrea glomerata* larvae reared at ambient and elevated CO_2_. Shell length measured at 2 d intervals from days 1–19 (mean ± SE) (24°C, salinity 34.5 ppt).

There was a significant effect of elevated CO_2_ and transgenerational exposure, on the shell length of *S*. *glomerata* larvae, but no interaction (Fig 3d). Exposure to elevated CO_2_ for 19 d caused a reduction in the size of larvae from both F2 lines (Figs 3d and 4; MS = 24584.33, F = 10.84, df = 1 x 8, *P*< 0.05). The effect of transgenerational exposure differed at the beginning and end of larval development. After 1 d in the experiment, the F2 *control* larvae were larger in size than the F2 CO_2_-exposed larvae at both ambient and elevated CO_2_ (MS = 1039.05, F = 789.56, df = 1 x 8, *P*< 0.001). By the end of larval development (19 d), however, this trend was reversed with the F2 *CO*_*2*_ *-exposed* larvae being larger in size than the F2 *control* larvae (Figs 3d and 4; MS = 33148.54, F = 14.61, df = 1 x 8, *P*< 0.01).

#### Shell length and heart rate of spat

**Fig 5:**
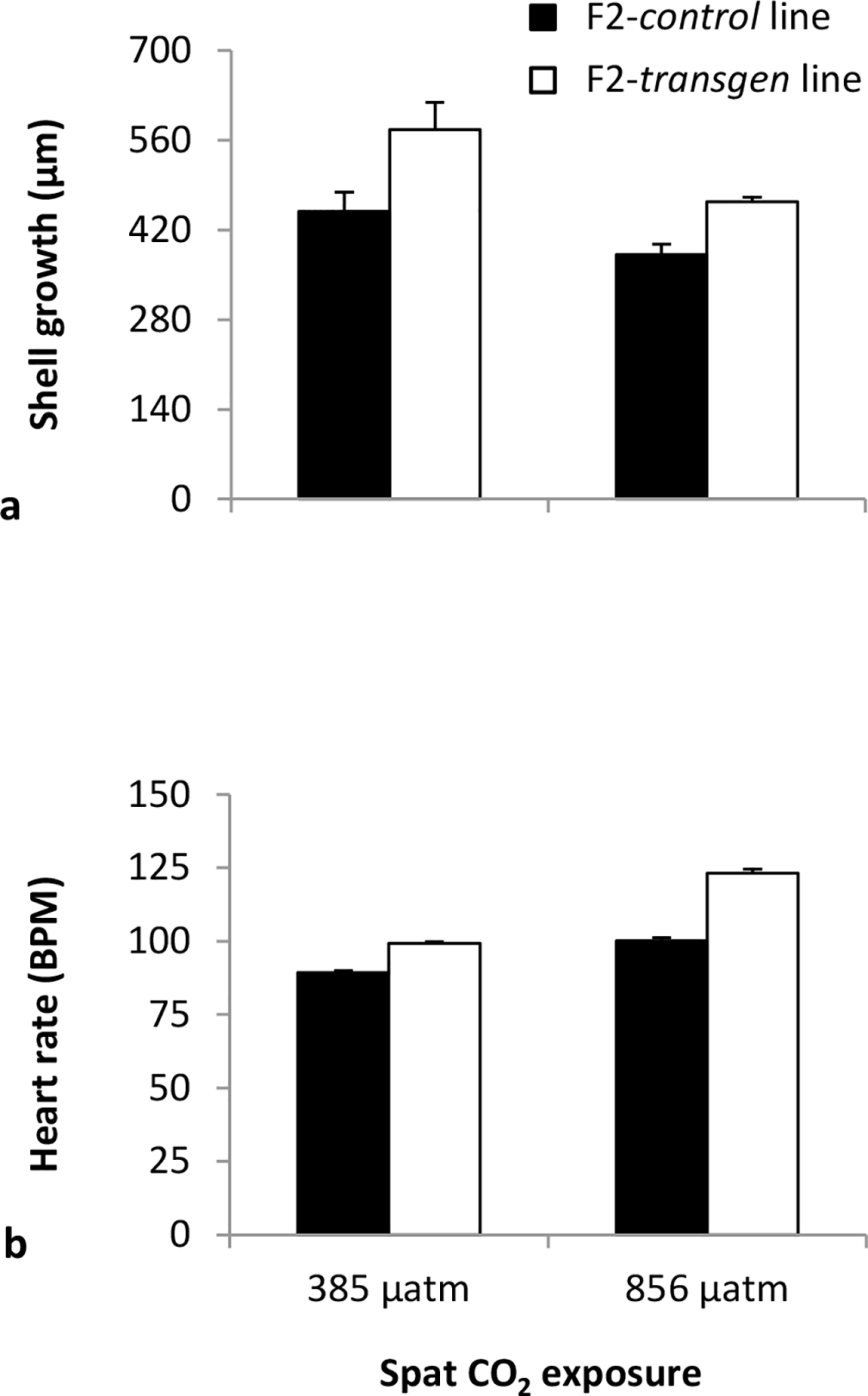
The response of F2-*control* and F2-*trangen Saccostrea glomerata* spat to ambient (385 μatm) and elevated (856 μatm) CO_2_. Shell growth **(a)**, and heart rate **(b)** following 6 d of exposure to the CO_2_ treatments (mean ± SE). Spat were 14 d post settlement at the beginning of the exposure (24°C, salinity 34.5 ppt).

Following settlement, there was once again a significant impact of elevated CO_2_ and transgenerational exposure but no interaction on the shell growth of spat (Fig 5a). After 6 d in the treatments, newly metamorphosed spat grew less at elevated (856 μatm) compared with ambient (385 μatm) CO_2_ (Fig 5a; MS = 24584.33, F = 10.84, df = 1 x 8, *P*< 0.05). In addition, the F2-*transgen* spat continued to perform better than the F2-*control* spat, growing faster at both ambient (385 μatm) and elevated (856 μatm) CO_2_ (Fig 5a; MS = 33148.54, F = 14.61, df = 1 x 8, *P*< 0.01).

Analysis of the heart rate of juvenile spat showed that it was significantly affected by the interaction of elevated CO_2_ and transgenerational exposure (Fig 5b). The heart rate (BPM) of both the F2-*control* and F2-*transgen* spat was increased following 6 d of exposure to elevated CO_2_ (Fig 5b; MS = 125.59, F = 39.35, df = 1 x 8; *P*< 0.001). Further, the heart rate of F2-*transgen* spat was greater than the F2-*control* spat at both ambient (385 μatm) and elevated (856 μatm) CO_2_.

## Discussion

Transgenerational exposure of adult Sydney rock oysters to elevated CO_2_ during reproductive conditioning was previously found to have positive carryover effects for the shell growth and development rate of larvae during exposure to the same level of elevated CO_2_ [5]. Here we tested whether these positive carryover effects that were found previously in F1 larvae could persist into adulthood (F1 adults) and whether subsequent transgenerational exposure of F1 adults to elevated CO_2_ would facilitate similar adaptive responses in the next generation (F2 larvae and juveniles). Overall, we found positive carryover effects, originating from the parental adults and F1 larvae following transgenerational exposure, persisted to improve the physiological performance of F1 adults at elevated CO_2_. F1-*transgen* adults had a greater capacity to regulate extracellular pH at elevated CO_2_ than the F1-*control* adults. In addition, subsequent transgenerational exposure of F1 adults led to an increase in the resilience of the larval and juvenile F2 offspring. F2-*transgen* larvae had a lower percentage abnormality at elevated CO_2_ than F2-*control* larvae and developed at a faster rate and were larger in size after 19 d at both ambient and elevated CO_2_. This trend continued following settlement with F2-*transgen* spat growing faster and having a faster heart rate (BPM) than F2-*control* spat at both ambient and elevated CO_2_. The results of our study show that transgenerational exposure of marine organisms to elevated CO_2_ may be even more beneficial than previously thought, as enhanced performance at elevated CO_2_ was found not only in F1 larvae following transgenerational exposure but also in F1 adults, despite the oysters spending a period of 14 mo at ambient CO_2_. Moreover, subsequent transgenerational exposure led to similar positive carryover effects in the F2 offspring.

The ability of marine organisms to maintain extracellular acid-base status during exposure to elevated CO_2_ has previously been identified as a critical physiological trait determining their level of resilience [32–34]. Reductions in extracellular pH during exposure to ocean acidification in particular can have serious consequences for marine species if left uncompensated, including reduced shell size [35–36] and somatic growth [37], changes in immune response [38], increased protein degradation [39], changes in SMR [5,37,39–42] and mortality in extreme cases [43–47]. Like most previous studies on shelled molluscs, exposure of *S*. *glomerata* adults in this study to elevated CO_2_ for 5 w led to a significant reduction in extracellular pH [34,39,40,48,49] (but not Marchant et al. [50]). The extent of this reduction was considerably less, however, in the F1-*transgen* adults compared to the F1-*control* adults, suggesting that previous transgenerational exposure of the parental adults and F1 larvae to elevated CO_2_ may have led to a greater capacity to regulate extracellular acid-base status.

The mechanisms of extracellular pH regulation in marine molluscs may involve passive buffering of free protons in the body fluids by either the CO_2_-bicarbonate system (albeit with limited efficiency) or the non-bicarbonate buffering system (such as N-terminal α amino acid groups of proteins or organic/ inorganic phosphate groups) [32]. Passive mechanisms minimize ongoing pH changes but do not defend steady state pH values [32]. Steady state pH values are set and maintained by active ion transport (active proton equivalent ion exchange across specialized epithelia causing the net accumulation of bicarbonate ions in the extracellular fluid) [33,51]. In this study, since the pH values recorded were steady state values, various degrees of passive buffering, for example by a greater concentration of non-bicarbonate buffers in the extracellular fluid of the F1-*transgen* adults, is considered unlikely for the greater regulation of extracellular pH at elevated CO_2_. Instead, it was expected that the enhanced efforts to compensate for the acidosis in the F1-*transgen* adults was an active process, incurring an increased metabolic cost [51]. Analysis of whole organism SMR, however, suggests that this did not occur (at least at the whole organism level), as the metabolic rate of F1 adults increased during exposure to elevated CO_2_ but this increase did not differ between the F1-*control* and F1-*transgen* adults. Instead, the F1-*transgen* adults may rather have shifted to some more energy efficient ion exchange mechanisms [51] or have undergone an adaptive alteration in their energy budget [37,40,52]. More energy may be diverted to extracellular acid-base regulation with less going into reproduction (as evidenced by the reduced size of the F2-*transgen* larvae at the beginning of larval development, 24 h post fertilization). Alterations in the partitioning of the energy budget of marine invertebrates during exposure to elevated CO_2_ have been suggested by a number of studies [5,37,40,52] but currently these alterations have been virtually unexplored.

The results of recent factorial breeding experiments suggest that increased maternal provisioning, whereby mothers invest more energy per egg/ offspring [53,54,55], may be one of the mechanisms immediately available to marine invertebrates species to safeguard their larvae against the impacts of elevated CO_2_ [8–9]. For example, maternal differences in larval size of the sea urchin *Strongylocentrotus franciscanus* and the mussel *Mytilus trossulus* were shown to directly influence the response of larvae to elevated CO_2_, with larger sized larvae being more resilient [8]. In our previous transgenerational experiment, maternal provisioning was identified as a possible mechanisms that caused increased resilience of larvae of *S*. *glomerata*. This was suggested as larvae from parents pre-exposed to elevated CO_2_ were larger in size at the beginning of larval development when compared to larvae from parents with no pre-exposure to elevated CO_2_ [5]. While maternal provisioning may indeed be a partial mechanism of resilience for *S*. *glomerata*, it does not explain why the F1-*transgen* adults in this study had a better capacity to regulate extracellular pH. In addition, the F2-*transgen* larvae were smaller in size at the beginning of larval development (1 d post-fertilization) yet still outperformed the F2-*control* larvae in all parameters measured in this study (growth, development rate, abnormality) at both ambient and elevated CO_2_. This brings us to the conclusion that epigenetic and/or genetic adaptive responses may also have been involved.

Epigenetic inheritance was identified as a mechanism for acclimation following transgenerational exposure of the anemonefish, *Amphiprion melanopus* [10] and sea urchin, *Strongylocentrotus droebachiensis* [13] to elevated CO_2_. In both species, pre-exposure of adults to elevated CO_2_ resulted in offspring that were not negatively affected by elevated CO_2_. Analysis of hatching (*A*. *melanopus*) and egg size (*S*. *droebachiensis*) revealed no significant difference between adult treatments, indicating that increases in maternal provisioning did not occur [10,13]. The authors instead suggested the occurrence of transgenerational epigenetic inheritance, where a beneficial change in gene expression in adults during exposure to elevated CO_2_ was passed to their offspring, improving offspring performance at elevated CO_2_ [10]. In this study, persistent epigenetic changes in gene expression, for example changes in the expression of key enzymes involved in acid-base regulation (e.g. Na^+^-K^+^-ATPase), may have occurred. These epigenetic changes may have originated during adult reproductive conditioning (transgenerational epigenetic inheritance) or F1 larval development and persisted to improve the performance of the F1 adults and F2 larvae and juvenile spat. Studies on other organisms have shown that the early life-history stages are critical developmental windows for lasting epigenetic changes during exposure to environmental factors. For example, in humans, maternal diet during pregnancy can lead to epigenetic changes in the embryo [56–58]. Further, in fish, environmental temperature during larval development can influence gender [59]. In the oyster, *Crassostrea gigas*, DNA methylation, a major mode of epigenetic change, has been suggested as a pivotal mechanism allowing them to survive in changing environments [60], however, the persistence of epigenetic change in oysters over multiple generations is yet to be demonstrated [61].

A final and perhaps most probable mechanism of adaptation of *S*. *glomerata* as measured in this study was through natural selection of genotypes that were more tolerant to elevated CO_2_ [62]. We used a group spawning design where spermatozoa from 10 males and eggs from 10 females were pooled together and allowed to fertilize. A result of this design is a diversity of genetic crosses. High mortality during larval development (46% in both the F1 and F2 generation; see Parker et al. [5] for F1 larval survival) may well have resulted in the survival of the most CO_2_ tolerant individuals. Indeed, in the calanoid copepod *Pseudocalanus acuspes*, natural selection due to high larval mortality (>50%) following transgenerational exposure to elevated CO_2_ was identified a possible mechanism associated with improved performance of adult fecundity at elevated CO_2_ [17].

Evidence from previous studies suggest that rapid evolutionary adaptation to ocean acidification may be possible (Sea urchin, Pespeni et al. [62]; Polychaete, Calosi et al. [63]). Pespeni et al. [62] found CO_2_-induced changes in allele frequencies related to lipid metabolism, ion homeostasis, protein modification and cell signalling in larvae of the purple sea urchin, *Strongylocentrotus purpuratus* that allowed them to maintain normal growth and development under elevated CO_2_ of 900 μatm. These changes in allele frequency were shown to be induced by natural selection during larval development. In the present study, natural selection seems to have favoured individuals with faster rates of growth. In the F2-*transgen* offspring, for example, shell growth of both larvae and juvenile spat was increased when compared to F2-*control* offspring. This increased growth occurred when offspring were reared in both the ambient and elevated CO_2_ treatments. Previous research on adult *S*. *glomerata* found that oysters which had been selectively bred by industry for faster growth were more resilient than wild oyster populations to elevated CO_2_ [5,26]. This resilience was attributed to their faster metabolic rate [5], a phenomenon that has also been observed in organisms with a natural resilience to elevated CO_2_ [33]. Faster growth rates of F2 larvae and juvenile spat found in this study following transgenerational exposure to elevated CO_2_ over two consecutive generations may also reflect an increase metabolic rate. In the F2-*transgen* juvenile spat for example, heart rate, a possible indicator of metabolic rate, was increased compared to the F2-*control* juvenile spat at both ambient and elevated CO_2_, suggesting that natural selection of individuals with an increase in metabolic rate potentially occurred [64–65]. While increased metabolic rate may positively influence the resilience of oysters to elevated CO_2_, it may negatively impact them in the in the presence of other environmental stressors [4,66–68]. For example, increased metabolic rate is predicted to narrow the thermal tolerance windows of marine invertebrates by decreasing aerobic scope [66]. Increased metabolic rate also increases an organism’s demand for food. If environmental food supply is not optimal, larvae and juvenile spat may be energy deficient, resulting in decreased growth, survival and metamorphic ability [19,68,69–71].

Oysters are sessile organisms which lack an escape response. Acclimation or adaptation of these species to elevated CO_2_ will therefore be essential in order to prevent local extinction over this century. Understanding the persistence of carryover effects on a wider range of phenotypic traits both within and across generations, the mechanisms underlying them and whether or not there are any limitations or trade-offs associated [20], will ultimately lead to a more reliable prediction of the impacts of near-future ocean acidification for marine species.
